# Effects of Jack Mackerel Meal Inclusion in Diets Replacing Fish Meal With Combined Tuna By‐Product and Corn Gluten Meals on the Growth and Feed Utilization of Red Sea Bream (*Pagrus major*), and Economic Efficiency

**DOI:** 10.1155/anu/4014545

**Published:** 2026-05-30

**Authors:** Jabed Hasan, Sung Hwoan Cho, Hee Sung Kim

**Affiliations:** ^1^ Department of Convergence Interdisciplinary Education of Maritime and Ocean Contents, National Korea Maritime and Ocean University, Busan Metropolitan City, 49112, Republic of Korea, kmou.ac.kr; ^2^ Division of Convergence on Marine Science, National Korea Maritime and Ocean University, Busan Metropolitan City, 49112, Republic of Korea, kmou.ac.kr; ^3^ Department of Marine Biology and Aquaculture, Gyeongsang National University, Tongyeong, 53064, Republic of Korea, gnu.ac.kr

**Keywords:** combined protein sources, feed enhancer, fish meal substitute, low-fish meal diet, sustainable aquaculture

## Abstract

A 56‐day feeding trial was conducted to investigate the effects of substituting fish meal (FM) with combined tuna by‐product meal and corn gluten meal (TC) at substitution levels of 25% and 50%, with or without 24% jack mackerel meal (JMM), on the growth, feed consumption (FC), feed utilization, blood chemistry, biochemical composition, and economic efficiency of red sea bream (*Pagrus major*). A total of 375 juveniles were allocated to 15, 50‐L flow‐through tanks (25 fish per tank) to evaluate five experimental diets that were formulated to be isoproteic and isolipidic in a triplicate group. A two‐way ANOVA [FM substitution levels (FMSL, 25% and 50%) × JMM inclusion (without and with)] experimental design was implemented. The control diet (Con) was comprised of 60% FM. In the Con diet, 25% and 50% of FM were substituted with TC, without and with JMM inclusion at these levels, designated as the TC25, TC50, TC25J, and TC50J, respectively. Fish were hand‐fed to apparent satiation. The 25%‐FM substitution diets resulted in significantly (*p* < 0.002 and *p* < 0.005, respectively) higher weight gain (WG) and specific growth rate (SGR) than the 50%‐FM substitution diets. Furthermore, the low‐FM diets with JMM inclusion yielded a significantly (*p* < 0.015 and *p* < 0.005, respectively) higher WG and SGR than those without JMM inclusion. The TC25J diet led to significantly (*p* < 0.011) higher WG than the Con and TC50 diets. The 25%‐FM substitution diets led to significantly (*p* < 0.002) higher FC than the 50%‐FM substitution diets. However, feed utilization, biological indices, proximate composition, and amino acid profiles of fish were not significantly (*p* > 0.05) affected by either FMSL or JMM inclusion. The 25%‐FM substitution diets led to significantly (*p* < 0.048) higher total monounsaturated fatty acid (∑MUFA) in the whole‐body fish than the 50%‐FM substitution diets. The 25%‐FM substitution diets also led to significantly (*p* < 0.011) higher economic profit index (EPI) than the 50%‐FM substitution diets. In conclusion, TC exhibits the potential to replace up to 50% of FM in the diet of red sea bream, regardless of JMM inclusion, without adversely impacting WG, SGR, FC, feed utilization, biological indices, proximate composition, amino acid profile, and economic sustainability. However, the TC25J diet is recommended as the most effective strategy for achieving optimal growth performance and FC and maximum EPI.

## 1. Introduction

Global aquatic animal production reached a record of 185.4 million metric tons (MT) in 2022, comprising 94.4 million MT from aquaculture (51%) and 91 million MT from capture fisheries (49%), with an average annual growth rate of 3.2%. Of the total production, 62% originated from marine areas, of which 31% was contributed by aquaculture, while 38% came from inland waters, with aquaculture accounting for 84% [1]. The estimated total first‐sale value of global aquatic animal production was USD 452 billion, with aquaculture accounting for USD 296 billion. Between 2020 and 2022, global farmed aquatic animal production increased by 6.7 million MT (7.6%), of which 5.9 million MT (87.9%) was attributed to Asia, primarily driven by finfish aquaculture (58.1%). Sea breams and porgies accounted for 0.9% of the global production of significant aquaculture species among finfish [1]. The sea bream market is expected to attain a value of USD 2451 million by 2035, increasing from USD 980.2 million, which corresponds to a compound annual growth rate of 9.8% during this period [2]. The red seabream (*P. major*) belongs to the Sparidae family within the Perciformes order and is a significant species in global aquaculture, especially in Eastern Asia and the Mediterranean [3]. Eastern Asian countries have a high demand for this seafood commodity, which is considered one of the most commercially important marine finfish species in Korea and Japan. In Korea, red sea bream production was approximately 6474 metric tons in 2024, which is corresponding to 8% of the total marine fish production (81,911 metric tons), and its market value was estimated to be 10.05 USD [1 USD = 1421.62 KRW] per kilogram of market‐sized fish [4]. For optimal growth performance, dietary protein and lipid requirements for juvenile red sea bream were reported to be 52% and 15%, respectively [5]. In general, carnivorous fish necessitate higher protein levels in their diets, and the quality of diets depends on the protein sources, comprising up to 60% of the total feed cost [6]. The future of the aquaculture industry is contingent upon the sustainable production of aquafeeds, which is a prerequisite for successful aquaculture production [7].

Fish meal (FM) serves as a prevalent protein source in aquafeeds, attributed to its high nutritional value [8, 9], balanced essential amino acids (EAAs), excellent digestibility, and palatability [10, 11], notwithstanding its cost. The volume of global capture fisheries converted into FM and fish oil has decreased in recent decades. In 1994, their utilization reached a peak of over 30 million MT, declining to over 17 million MT by 2022 [1]. The demand for FM is surging due to the rapid expansion of the aquaculture industry, alongside requirements from pig and poultry farming, as well as the pet food and pharmaceutical sectors. In 2021, more than 87% of FM was utilized in aquaculture [12]. The increasing demand for FM and fish oil has led to higher prices, which have adversely affected their utilization in aquaculture, resulting in a reduction in their inclusion in grower diets [1]. FM is becoming an economically unfeasible and environmentally unsustainable option for aquafeed production [7]. The decrease is linked to the utilization of alternative protein sources, including animal by‐products and plant‐based ingredients, which have improved feeding efficiency and helped mitigate the impacts of supply and price volatility. Numerous studies have effectively evaluated different animal and plant protein sources as substitutes for FM in red sea bream diets, demonstrating substantial results [13–18].

Individual animal and plant protein sources show potential as an FM substitute; however, their standalone application is often limited by inherent deficiencies in EAAs [19]. Addressing these deficiencies remains a major challenge in FM substitution in aquafeeds, especially given the ongoing debates regarding the efficacy of supplementing diets with synthetic amino acids [20–22]. Combining animal and plant protein sources has generally proven more effective for FM replacement than relying on a single protein source [15]. This approach can improve the balance of amino acids, reduce anti‐nutritional factors commonly found in plant ingredients, and enhance growth and health in aquaculture species. Consequently, combining animal and plant proteins may better rectify nutritional imbalances compared to exclusive dependance on synthetic amino acid supplementation [19, 23, 24]. Moreover, the use of combined protein sources proves to be more economically feasible, enabling a higher level of FM replacement compared to individual protein sources alone [25, 26].

Tuna by‐products derived from the processing of skipjack and yellowfin tuna represent fishery by‐products associated with the canning industry [27, 28]. Canning tuna results in the estimated loss of 50%–70% of the entire fish [29]. It may serve as a potential alternative protein source to FM in aquafeeds. Fishery by‐products contain high‐quality protein, lipids, and essential micronutrients, including vitamins A, B_2_, B_3_, and D, as well as minerals including iron, zinc, selenium, and iodine [30]. Studies indicate that tuna by‐product meal (TBM) can serve as a partial substitute for FM in the diets of olive flounder (*Paralichthys olivaceus*) [28], spotted rose snapper (*Lutjanus guttatus*) [31], rockfish (*Sebastes schlegeli*) [32], and red sea bream [33, 34]. Particularly, Baek and Cho [34] recommended 40% of FM substitution with TBM in the red sea bream diet when fish were fed with a 55% FM‐based diet or one of the diets replacing 20%, 40%, 60%, 80%, and 100% of FM were replaced with TBM in the 8‐week feeding trial.

Corn gluten meal (CGM) is a high‐protein by‐product from corn starch production [35] and serves as a commonly utilized plant protein source to replace FM in aquafeeds [36]. CGM is a nutrient‐rich, high‐protein feed ingredient, abundant in vitamins and most EAA, although it has limitations in arginine and lysine content [36–38]. CGM has been utilized as an FM alternative in the diets of several carnivorous fish species, including barramundi (*Lates calcarifer*) [36], cobia (*Rachycentron canadum*) [39], olive flounder [40], Japanese seabass (*Lateolabrax japonicas*) [41], rockfish [42], puffer (*Takifugu fasciatus*) [38], spotted rose snapper [37], and turbot (*Scophthalmus maximus*) [43]. Particularly, Kwon and Cho [44] recommended 20% of FM replacement with CGM in a 55% FM‐based diet of red sea bream or one of the diets replacing 20%, 40%, and 60% of FM with CGM in the 8‐week feeding trial.

The extensive substitution of alternative ingredients in aquafeeds often results in reduced palatability and decreased feed consumption (FC), ultimately compromising growth performance in fish [24, 45]. The inclusion of a feed enhancer or protein ingredient with high attractant properties in low‐FM diets has the potential to improve diet palatability and stimulate feed intake in target fish species [26, 45–48]. Among 18 protein sources, red sea bream exhibited the highest attraction behavior to jack mackerel meal (JMM), and replacing 40% of FM with JMM significantly improved FC and growth [49]. Thus, incorporating JMM into the low‐FM diets can enhance FC and growth in red sea bream, offering a sustainable strategy for effective aquaculture practices.

To the best of our knowledge, limited research has investigated the combined effects of animal and plant protein sources as a replacement for FM in the diets of red sea bream. Therefore, the present study was conducted to evaluate the effects of combined TBM and CGM, as an alternative to FM, with or without JMM, on growth, FC, feed utilization, blood chemistry, biochemical composition, and economic efficiency in red sea bream.

## 2. Materials and Methods

### 2.1. Experimental Design

Similar‐sized juveniles of red sea bream were purchased from a private hatchery (Tongyeong‐si, Gyeongsangnam‐do, Korea) to the experimental unit (Institute of Ocean Science Education, Gangneung‐Wonju National University, Gangneung‐si, Gangwon‐do, Korea) following a standard procedure. Firstly, fish were acclimatized for 14 days in a circular flow‐through tank having a capacity of 5 tons filled with a water volume of 3.5 tons. During the acclimatization period, fish were supplied with commercial extruded pellets (National Federation of Fisheries Cooperatives Feed, Uiryeong‐gun, Gyeongsangnam‐do, Korea) comprising crude protein (CP, 50%) and crude lipid (CL, 13%), administered twice daily at 2%–3% total biomass. Following, 375 randomly selected juveniles (initial weight 2.0 ± 0.02 g; mean ± SE) were allocated to 15 rectangular flow‐through tanks (25 random fish per tank; in triplicate), each containing a water volume of 40 L (tank capacity 50 L). The mixture of underground and sand‐filtered seawater at a ratio of 1:1 was supplied into each tank with a flow rate of 4.5 L/min, and aeration was sufficiently supplied to each tank constantly. Besides, the natural photoperiod condition was maintained. Water quality parameters, including dissolved oxygen, pH, salinity, and temperature, were assessed using a digital multimeter (AZ‐8603, Taichung, Taiwan) and found to be 7.7 ± 0.23 mg/L (mean ± SD), 7.6 ± 0.10, 30.7 ± 0.38 g/L, and 20.1 ± 1.73°C, respectively. Siphon cleaning was conducted regularly at the bottom of each tank, and the dead fish were promptly removed upon detection to ensure proper water quality throughout the experimental period.

### 2.2. Preparation of the Experimental Diets and Feeding

For the experimental design, a two‐way ANOVA (FM substitution levels [FMSL, 25% and 50%] × JMM inclusion [without and with]) was performed to test the efficacy of the FM substitution level and JMM inclusion in low‐FM diets. The feed formulation and proximate composition of the experimental diets are presented in Table 1. A total of five isoproteic (52.1%) and isolipidic (15.2%) diets were formulated to meet the nutritional requirements for red sea bream [5], with each diet supplied in triplicated groups of tanks. The control (Con) diet consisted of FM (60%) and defatted soybean meal (13.5%) as the primary protein sources. Fish (7.0%) and soybean oils (2.5%) served as the sources of lipid, while wheat flour (14.5%) served as the source of carbohydrate. In the Con diet, 25% and 50% of FM were substituted with combined tuna by‐product meal and corn gluten meal (TC), designated as TC25 and TC50, respectively. The TC consisted of a combination of TBM and CGM at a ratio of 1:1, and each ingredient was acquired from local traders. Additionally, 24% JMM was incorporated into all TC‐substituted diets [49] at the expense of FM, designated as TC25J and TC50J, respectively.

**Table 1:** Ingredients and chemical composition of the experimental diets (%, dry matter basis).

	Experimental diets				
	Con	TC25	TC50	TC25J	TC50J
Ingredients (%)					
Fish meal^a^	60.0	45.0	30.0	21.0	6.0
Combined tuna by‐product and corn gluten meal^b^	—	17.8	35.6	17.8	35.6
Jack mackerel meal^c^	—	—	—	24.0	24.0
Defatted soybean meal	13.5	13.5	13.5	13.5	13.5
Wheat flour	14.5	11.8	9.1	12.2	9.5
Fish oil	7.0	7.0	7.0	7.0	7.0
Soybean oil	2.5	2.4	2.3	2.0	1.9
Vitamin premix^d^	1.0	1.0	1.0	1.0	1.0
Mineral premix^e^	1.0	1.0	1.0	1.0	1.0
Choline	0.5	0.5	0.5	0.5	0.5
Nutrients (%)					
Dry matter	97.1	97.5	97.1	97.6	97.3
Crude protein	52.1	52.0	52.2	51.8	52.2
Crude lipid	15.1	15.3	15.3	15.3	15.1
Ash	11.2	11.2	11.5	11.1	11.3
Carbohydrate^f^	21.6	21.4	21.0	21.8	21.4
Gross energy (kcal/g)	5.1	5.1	5.2	5.1	5.1

^a^Fish meal (FM; crude protein [CP]: 72.2%, crude lipid [CL]: 8.1%, and ash: 15.1%) was imported from Chile (USD 2.25/kg FM, USD 1 = 1421.62 KRW).

^b^Combined tuna by‐product meal and corn gluten meal (TC; CP, 62.8%, CL, 7.68%, and ash: 12.07%) was the combination of tuna by‐product meal (TBM, CP, 54.5%, CL, 14.46%, and ash: 21.65%) and corn gluten meal (CGM; CP, 71.1%, CL, 0.9%, and ash: 2.5%) at the ratio of 1:1, and each ingredients was purchased from Woojin Feed Ind. Co. Ltd., (Incheon Metropolitan City, Korea) (USD 1.20/kg TBM) and Hyunjin Livestock Distribution Co. Ltd. (Incheon Metropolitan City, Korea) (USD 0.74/kg CGM), respectively.

^c^Jack mackerel meal (JMM; CP, 73.0%, CL, 9.8%, and ash: 13.6%) was imported from Chile (USD 2.67/kg JMM).

^d^Vitamin premix (g/kg mix): L, ascorbic acid; 121.2; DL‐α‐tocophyryl acetate, 18.8; thiamin hydrochloride, 2.7; riboglavin, 9.1; pyridoxine hydrochloride, 1.8; niacin, 36.4; Ca‐D, pantothenate; 12.7; myo‐inositol, 181.8; D, biotin; 0.27; folic acid, 0.68; p‐aminobenzoic acid, 18.2; menadione, 1.8; retinyl acetate, 0.73; cholecalciferol, 0.003; cyanocobalamin, 0.003.

^e^Mineral premix (g/kg mix): MgSO_4_·7H_2_O, 80.0; NaH_2_PO_4_·2H_2_O, 370.0; ferric citrate, 40.0; ZnSO_4_·7H_2_O, 20.0; Ca‐lactate, 356.5; CuCl, 0.2; AlCl_3_·6H_2_O, 0.15; KI, 0.15; Na_2_Se_2_O_3_, 0.01; MnSO_4_H_2_O, 2.0; CoCl_2_·6H_2_O, 1.0.

^f^Carbohydrate was calculated by the difference [Carbohydrate = 100 − (CP + CL + ash)].

To prepare the experimental diets, initially, the ingredients were well mixed with water at a 3:1 ratio using a vertical mixer (B20GA, Eben Commerce Korea, Ansan‐si, Gyeonggi‐do, Korea). The final dimension of the diets was formulated at 3 mm in diameter using a pelleting machine (SMC‐32; SL Company, Incheon Metropolitan City, Korea). Ultimately, the diets were dried in a drying machine (JW‐1350ED, Jinwoo Electronics Co., Ltd., Hwaseong‐si, Gyeonggi‐do, Korea) at 45°C for 24 h and subsequently kept in a laboratory freezer at −20°C for further use. The fish were hand‐fed to apparent satiation twice (08:00 and 17:00) a day throughout the feeding trial.

### 2.3. Assessment of Performance of Red Sea Bream

Following a 56‐day feeding trial, fish were starved for 24 h before being anesthetized with 50 mg/L tricaine methanesulfonate (MS‐222). Subsequently, the survival and weight gain (WG) of each fish in each tank were evaluated by counting and weighing them in their entirety. The biological indices, such as the specific growth rate (SGR), viscerosomatic index (VSI), hepatosomatic index (HSI), and condition factor (CF) were determined using randomly selected 10 anesthetized fish from each tank as the follows: SGR (%/day) = (Ln final weight of fish (g) − Ln initial weight of fish (g)) × 100/days of the feeding trial; CF (g/cm^3^) = body weight of fish (g) × 100/total length of fish (cm)^3^; VSI (%) = viscera weight of fish (g) × 100/body weight of fish (g); and HSI (%) = liver weight of fish (g) × 100/body weight of fish (g). In addition, feed efficiency (FE), protein efficiency ratio (PER), and protein retention (PR) were calculated by using the following formulas: FE = (total final weight of fish (g) − total initial weight of fish (g) + total weight of dead fish (g))/total FC (g); PER = WG of fish (g)/protein consumption of fish (g); and PR (%) = protein gain of fish (g) × 100/protein consumption of fish (g).

### 2.4. Assessment of Blood Biochemistry of Red Sea Bream

Blood samples were collected from the caudal vein of five randomly selected anesthetized fish from each tank using a 1 mL heparinized syringe for analysis. The plasma samples were obtained by centrifuging the blood samples at a speed of 2700×*g* at 4°C for 10 min and stored in a deep freezer at −70°C for subsequent analysis. Later, an automated chemistry analyzer (FUJI DRI‐CHEM NX500i, Fujifilm Corp., Tokyo, Japan) was utilized to determine the plasma parameters, including aspartate aminotransferase (AST), alanine aminotransferase (ALT), alkaline phosphatase (ALP), total bilirubin (T‐BIL), total cholesterol (T‐CHO), triglyceride (TG), total protein (TP), and albumin (ALB). Serum samples were obtained in the same manner as the plasma samples (using a syringe instead of a syringe pre‐coated with heparin) and subsequently assessed for lysozyme (LYZ) activity and superoxide dismutase (SOD) according to the previously established methods [50, 51].

### 2.5. Assessment of the Biochemical Composition of the Experimental Feeds and Fish

The main protein sources (FM, JMM, and TC), all experimental diets, initial juvenile fish (*n* = 10) before the experiment, and remaining (≥13) fish from each tank were homogenized for the proximate composition analysis according to the standard method [52]. The moisture content of the samples was determined through an oven (LDO‐250F, Dalhan Labtech Co., Ltd., Namyangju‐si, Gyeonggi‐do, Korea) at 105°C for 6 h for dry samples and 24 h for wet samples. The Kjeldahl method (KjeltecTM2100, Distillation Unit, Foss, Hillerød, Denmark) and the ether‐extraction method (SoxtecTM 2043, Fat Extraction System, Foss, Hillerød, Denmark) were utilized to assess the crude protein and crude lipid content, respectively. To determine the ash content, a muffle furnace (MF‐14, Han Yang Scientific Equipment Co., Ltd., Seoul Special City, Korea) was operated at 550°C for 4 h.

An automated amino acid analyser (L‐8800, Japan) was deployed to examine the amino acid profiles, excluding tryptophan, in all samples. For instance, 30 mL of 6 N hydrochloric acid was added after placing 0.2 g of the sample in a decomposition tube. Nitrogen gas was subsequently introduced and hydrolyzed at 110°C for a duration of 24 h. A 0.45 µm nylon syringe filter was used to filter the concentrated filtrate prior to analysis. Furthermore, tryptophan content of the samples was measured using high‐performance liquid chromatography (S1125 HPLC, Sykam GmbH, Eresing, Germany).

The fatty acid profiles were ascertained by contrasting the experimental samples to a recognized standard (FAME mix CRM47885, Supelco, St. Louis, MO, USA). Lipids were extracted from the samples using a 2:1 (v/v) mixture of methanol and chloroform accordingly [53]. A gas chromatograph (HP6890, Agilent Technologies Inc., Santa Clara, CA, USA) fitted with an SPTM‐2560 capillary column (inner diameter 100 m × 0.25 mm and film thickness 0.20 µm (SupelcoTM, St. Louis, MO, USA) was used to quantify the extracted lipids after they had been methylated with 14% BF‐3MeOH. Each fatty acid peak was identified by comparing it to the methyl ester of the reference fatty acid.

### 2.6. Economic Analysis of the Study

An economic analysis, viz., economic conversion ratio (ECR) and economic profit index (EPI), was conducted using the following formulas [54]: ECR (USD/kg) = FC (kg/fish) / WG (kg/fish) × diet price (USD/kg); and EPI (USD/fish) = [final weight (kg/fish) × fish sale price (USD/kg)] − [ECR (USD/kg) × WG (kg/fish)]. The cost of each component was multiplied by the composition of the diets to ascertain the price. The price of each component, such as FM, TC, JMM, defatted soybean meal, wheat flour, fish oil, soybean oil, vitamin premix, mineral premix, and choline, was 2.25, 1.94, 2.67, 0.60, 0.47, 2.42, 1.57, 728, 5.85, and 1.27, respectively, in USD/kg. The estimated price of the juvenile red sea bream was USD 100 per kg of fish.

### 2.7. Statistical Analysis

Statistical analysis was performed using SPSS version 25.0 (IBM SPSS Statistics for Windows, IBM Corp., Armonk, NY, USA). Before employing statistical analysis, means of all parameters were checked for homogeneity of variances and normality test through Levene’s test and Shapiro‐Wilk test, respectively, and found no violations (*p* > 0.05). Additionally, all percentage data were transformed to arcsine format before statistical analysis. To evaluate the effects of FM substitution, inclusion of JMM, and their interaction, a two‐way ANOVA was conducted at a statistical level of *p* < 0.05, followed by one‐way ANOVA to compare the means of the experimental diets. Duncan’s multiple range test was performed as a post‐hoc analysis if statistical differences (*p* < 0.05) were found among dietary treatments. The principal component analysis (PCA) and different plots were analyzed and generated using OriginPro 2025 software (Northampton, MA, USA).

## 3. Results

### 3.1. Amino Acid and Fatty Acid Profiles of the Main Protein Ingredients and Experimental Diets

In the major protein ingredients, TC exhibited higher leucine and phenylalanine, and alanine, glutamic acid, and proline content compared to FM across all EAA and non‐essential amino acid (NEAA), respectively (Table 2). JMM retained higher histidine and glycine values compared to FM among all the EAA and NEAA, respectively. In the experimental diets, leucine and phenylalanine tended to increase, corresponding to increased FM substitution in the low‐FM diets, similar to the trends observed in NEAA for alanine, glutamic acid, and proline. Furthermore, JMM inclusion in the low‐FM diets led to a lowering trend in all EAA, except for histidine and NEAA, except for glutamic acid.

**Table 2:** Amino acid profiles (% of the diet) of the major protein ingredients and the experimental diets.

Parameters	Major protein ingredients			Requirement	Experimental diets				
	FM	JMM	TC		Con	TC25	TC50	TC25J	TC50J
Essential amino acids (EAA)									
Arginine	4.24	4.02	2.49	2.37^a^	2.88	2.79	2.70	2.67	2.64
Histidine	2.00	2.07	1.37	—	1.34	1.27	1.19	1.29	1.25
Isoleucine	3.02	2.70	2.21	—	2.09	1.85	1.80	1.76	1.70
Leucine	5.47	5.01	7.27	—	3.76	4.21	4.47	4.15	4.37
Lysine	5.80	5.48	2.33	1.79^b^	3.84	3.37	2.95	3.28	2.90
Phenylalanine	2.93	2.62	3.02	—	2.17	2.18	2.19	2.07	2.13
Threonine	3.14	2.98	2.21	—	2.19	2.00	1.88	1.97	1.81
Tryptophan	0.54	0.53	0.32	—	0.42	0.41	0.39	0.38	0.36
Valine	3.61	3.22	2.59	0.90^c^	2.54	2.39	2.35	2.31	2.25
∑EAA^d^	30.75	28.63	23.80	—	21.23	20.47	19.92	19.88	19.41
Non‐essential amino acids (NEAA)									
Alanine	4.59	4.42	4.64	—	2.95	3.17	3.21	3.10	3.18
Aspartic acid	6.65	6.21	4.21	—	4.73	4.60	4.33	4.53	4.28
Glutamic acid	9.29	8.70	10.12	—	7.18	7.27	7.53	7.30	7.49
Glycine	4.34	4.39	2.81	—	2.89	2.83	2.58	2.85	2.55
Proline	3.00	2.97	4.48	—	2.24	2.43	2.67	2.38	2.57
Serine	2.90	2.73	2.74	—	2.11	2.00	1.87	1.94	1.82
Tyrosine	2.30	1.83	2.22	—	1.50	1.26	1.22	1.20	1.15
∑NEAA^e^	33.07	31.25	31.20	—	23.60	23.56	23.41	23.30	23.04

*Note:* TC: combined tuna by‐product meal and corn gluten meal (1:1); Con: the 60% FM basal diet; TC25: dietary substitution of 25%‐FM with TC; TC50: dietary substitution of 50%‐FM with TC; TC25J, dietary substitution of 25%‐FM with TC with JMM inclusion; TC50J, dietary substitution of 50%‐FM with TC with JMM inclusion.

Abbreviations: FM, fish meal; JMM, jack mackerel meal.

^a^Arginine [55].

^b^Lysine [56].

^c^Valine [57].

^d^∑EAA, total essential amino acids.

^e^∑NEAA, total nonessential amino acids.

TC had lower total saturated fatty acid (∑SFA), total monounsaturated fatty acid (∑MUFA), and n‐3 highly unsaturated fatty acid (∑n‐3 HUFA) compared to FM; on the other hand, JMM had a higher ∑MUFA (Table 3). The ∑SFA, ∑MUFA, and ∑n‐3 HUFA exhibited a decreasing trend with an increased FM substitution; however, at the same FM substitution level with JMM inclusion in the low‐FM diets, ∑MUFA showed an increasing trend with higher FM substitution, while ∑SFA and ∑n‐3 HUFA showed a decreasing trend.

**Table 3:** Fatty acid profiles (% of total fatty acids) of the major protein ingredients and the experimental diets.

Parameters	Major protein ingredients			Experimental diets				
	FM	JMM	TC	Con	TC25	TC50	TC25J	TC50J
C14:0	6.36	5.27	2.15	2.70	2.51	2.26	2.38	2.16
C16:0	21.40	19.40	19.43	14.83	14.66	14.56	14.35	14.27
C18:0	3.83	6.33	4.71	3.80	3.95	4.19	4.33	4.52
∑SFA^a^	31.59	31.00	26.28	21.33	21.12	21.01	21.06	20.95
C16:1n–7	7.04	7.06	2.81	3.10	3.02	2.74	3.06	2.75
C17:1n–7	1.13	1.05	0.57	0.50	0.46	0.41	0.45	0.42
C18:1n–9	16.44	21.41	21.26	21.82	22.30	22.84	23.41	24.46
C20:1n–9	3.49	1.50	0.57	2.69	2.33	2.08	2.22	1.97
C22:1n–9	0.16	0.21	0.37	0.30	0.32	0.34	0.32	0.36
C24:1n–9	3.25	3.64	0.50	1.38	1.09	0.90	1.19	0.97
∑MUFA^b^	31.51	34.87	26.06	29.79	29.52	29.31	30.65	30.93
C18:2n–6	4.88	2.29	28.91	29.27	30.40	31.03	30.14	30.89
C18:3n–3	0.36	0.63	1.45	3.49	3.77	3.81	3.87	3.96
C18:3n–6	0.29	0.25	0.53	0.55	0.63	0.65	0.60	0.63
C20:4n–6	2.04	2.51	1.10	2.02	1.97	1.93	2.00	1.96
C20:5n–3	14.96	11.60	2.46	6.37	5.31	4.36	4.55	3.90
C22:2n–6	0.53	0.62	0.45	0.32	0.30	0.29	0.35	0.33
C22:6n–3	9.06	12.11	9.07	4.25	4.27	4.30	4.37	4.43
DHA:EPA	0.61	1.04	3.69	0.67	0.80	0.99	0.96	1.14
∑n‐3 HUFA^c^	24.02	23.71	11.52	10.62	9.58	8.66	8.92	8.33
Unknown	4.78	4.12	3.71	2.61	2.71	3.31	2.41	2.02

*Note:* TC: combined tuna by‐product meal and corn gluten meal (1:1); Con: the 60% FM basal diet; TC25: dietary substitution of 25%‐FM with TC; TC50: dietary substitution of 50%‐FM with TC; TC25J, dietary substitution of 25%‐FM with TC with JMM inclusion; TC50J, dietary substitution of 50%‐FM with TC with JMM inclusion.

Abbreviations: DHA, docosahexaenoic acid; EPA, eicosapentaenoic acid; FM, fish meal; JMM, jack mackerel meal.

^a^∑SFA, total saturated fatty acids.

^b^∑MUFA, total monounsaturated fatty acids.

^c^∑n‐3 HUFA, total n‐3 highly unsaturated fatty acids.

### 3.2. Growth Performance of Red Sea Bream

Survival of red sea bream under different experimental diets ranged from 96.00% to 98.67% and it was not significantly (*p* > 0.282 for both) influenced by either FMSL or JMM inclusion (Table 4).

**Table 4:** Survival (%), weight gain (WG, g/fish), and specific growth rate (SGR, %/day) of red sea bream fed the experimental diets for 56 days.

Experimental diets	Initial weight (g/fish)	Final weight (g/fish)	Survival (%)	WG (g/fish)	SGR^a^ (%/day)
Con	2.03 ± 0.027	10.43 ± 0.186	97.33 ± 1.333	8.41 ± 0.159^bc^	2.93 ± 0.009^bc^
TC25	2.00 ± 0.046	10.67 ± 0.091	97.33 ± 1.333	8.67 ± 0.077^ab^	2.99 ± 0.036^b^
TC50	2.03 ± 0.027	10.19 ± 0.121	96.00 ± 0.000	8.17 ± 0.097^c^	2.88 ± 0.009^c^
TC25J	1.95 ± 0.027	10.80 ± 0.122	98.67 ± 1.333	8.85 ± 0.106^a^	3.06 ± 0.019^a^
TC50J	1.97 ± 0.027	10.52 ± 0.095	97.33 ± 1.333	8.55 ± 0.080^ab^	2.99 ± 0.019^b^
*p*‐Value	—	—	*p* > 0.655	*p* < 0.011	*p* < 0.001
Major effect: FMSL					
25%	—	—	98.00 ± 0.894	8.76 ± 0.071^A^	3.03 ± 0.024^A^
50%	—	—	96.67 ± 0.667	8.36 ± 0.102^B^	2.94 ± 0.025^B^
Major effect: JMM inclusion					
Without	—	—	96.67 ± 0.667	8.42 ± 0.126^B^	2.94 ± 0.029^B^
With	—	—	98.00 ± 0.894	8.70 ± 0.090^A^	3.02 ± 0.020^A^
Two‐way ANOVA					
FMSL	—	—	*p* > 0.282	*p* < 0.002	*p* < 0.005
JMM inclusion	—	—	*p* > 0.282	*p* < 0.015	*p* < 0.005
Interaction	—	—	*p* > 0.999	*p* > 0.305	*p* > 0.451

*Note:* Con: the 60% fish meal (FM) basal diet; TC25: dietary substitution of 25%‐FM with TC; TC50: dietary substitution of 50%‐FM with TC; TC25J, dietary substitution of 25%‐FM with TC with JMM inclusion; TC50J, dietary substitution of 50%‐FM with TC with JMM inclusion. Values (means of triplicate ± standard error) with different lowercase and uppercase letters indicate significant differences (*p* < 0.05) based on Duncan’s multiple range test and two‐way ANOVA, respectively.

Abbreviation: FMSL, fish meal substitution level.

^a^Specific growth rate (SGR, %/day) = [Ln final weight of fish (g) − Ln initial weight of fish (g)] × 100/days of the feeding trial.

The 25%‐FM substitution diets produced significantly (*p* < 0.002 and *p* < 0.005) higher WG and SGR (8.76 ± 0.071 g/fish and 3.03 ± 0.024%/day) in comparison to the 50%‐FM substitution diets (8.36 ± 0.102 g/fish and 2.94 ± 0.025%/day, respectively). Additionally, the low‐FM diets with JMM inclusion showed significantly (*p* < 0.015 and *p* < 0.005) higher WG and SGR (8.70 ± 0.090 g/fish and 3.02 ± 0.020%/day) compared to those without JMM inclusion (8.42 ± 0.126 g/fish and 2.94 ± 0.028%/day, respectively). In multiple comparisons, the TC25J diet produced significantly (*p* < 0.011) higher WG (8.85 ± 0.106 g/fish) than the Con (8.41 ± 0.159 g/fish) and TC50 (8.17 ± 0.097 g/fish) diets, but not significantly different from the TC25 (8.67 ± 0.077 g/fish) and TC50J (8.55 ± 0.080 g/fish) diets. The TC25J diet (3.06 ± 0.019%/day) also resulted significantly (*p* < 0.001) higher SGR than the Con (2.93 ± 0.009%/day), TC25 (2.99 ± 0.036%/day), TC50 (2.88 ± 0.009%/day), and TC50J (2.99 ± 0.019%/day) diets.

### 3.3. Feed Utilization and Biological Metrics of Red Sea Bream

The 25%‐FM substitution diets (8.56 ± 0.089 g/fish) showed significantly (*p* < 0.002) higher FC than the 50%‐FM substitution diets (8.04 ± 0.082 g/fish) (Table 5). Fish fed the TC25J diet (8.65 ± 0.169 g/fish) showed significantly (*p* < 0.014) higher FC than the Con (8.07 ± 0.183 g/fish), TC50 (7.92 ± 0.048 g/fish), and TC50J (8.17 ± 0.126 g/fish) diets, but not significantly different from fish fed the TC25 diet (8.47 ± 0.055 g/fish).

**Table 5:** Feed consumption (FC, g/fish), feed efficiency (FE), protein efficiency ratio (PER), protein retention (PR, %), condition factor (CF, g/cm^3^), viscerosomatic index (VSI, %), and hepatosomatic index (HSI, %) of red sea bream fed the experimental diets for 56 days.

Experimental diets	FC (g/fish)	FE^a^	PER^b^	PR^c^ (%)	CF^d^ (g/cm^3^)	VSI^e^ (%)	HSI^f^ (%)
Con	8.07 ± 0.183^bc^	1.03 ± 0.016	2.00 ± 0.026	32.17 ± 0.650	1.88 ± 0.017	6.53 ± 0.024	1.19 ± 0.038
TC25	8.47 ± 0.055^ab^	1.02 ± 0.002	1.97 ± 0.006	33.10 ± 0.360	1.89 ± 0.020	6.75 ± 0.108	1.29 ± 0.086
TC50	7.92 ± 0.048^c^	1.02 ± 0.016	1.98 ± 0.032	32.32 ± 0.288	1.85 ± 0.015	6.77 ± 0.061	1.32 ± 0.072
TC25J	8.65 ± 0.169^a^	1.02 ± 0.009	1.97 ± 0.016	32.68 ± 0.600	1.90 ± 0.022	6.61 ± 0.074	1.24 ± 0.070
TC50J	8.17 ± 0.126^bc^	1.04 ± 0.019	2.02 ± 0.032	32.52 ± 0.405	1.88 ± 0.015	6.60 ± 0.086	1.30 ± 0.044
*p*‐Value	*p* < 0.014	*p* > 0.544	*p* > 0.516	*p* > 0.684	*p* > 0.390	*p* > 0.199	*p* > 0.625
Major effect: FMSL							
25%	8.56 ± 0.089^A^	1.02 ± 0.004	1.97 ± 0.008	32.89 ± 0.326	1.90 ± 0.014	6.68 ± 0.066	1.26 ± 0.051
50%	8.04 ± 0.082^B^	1.03 ± 0.012	2.00 ± 0.022	32.42 ± 0.226	1.87 ± 0.012	6.68 ± 0.061	1.31 ± 0.038
Major effect: JMM inclusion							
Without	8.19 ± 0.127	1.02 ± 0.007	1.97 ± 0.014	32.71 ± 0.269	1.87 ± 0.014	6.67 ± 0.056	1.30 ± 0.051
With	8.41 ± 0.143	1.03 ± 0.010	2.00 ± 0.019	32.60 ± 0.326	1.89 ± 0.013	6.61 ± 0.051	1.27 ± 0.039
Two‐way ANOVA							
FMSL	*p* < 0.002	*p* > 0.328	*p* > 0.258	*p* > 0.298	*p* > 0.142	*p* > 0.985	*p* > 0.548
JMM inclusion	*p* > 0.088	*p* > 0.328	*p* > 0.371	*p* > 0.817	*p* > 0.241	*p* > 0.102	*p* > 0.643
Interaction	*p* > 0.773	*p* > 0.457	*p* > 0.371	*p* > 0.494	*p* > 0.727	*p* > 0.833	*p* > 0.889

*Note:* Con: the 60% fish meal (FM) basal diet; TC25: dietary substitution of 25%‐FM with TC; TC50: dietary substitution of 50%‐FM with TC; TC25J, dietary substitution of 25%‐FM with TC with JMM inclusion; TC50J, dietary substitution of 50%‐FM with TC with JMM inclusion. Values (means of triplicate ± standard error) with different lowercase and uppercase letters indicate significant differences (*p* < 0.05) based on Duncan’s multiple range test and two‐way ANOVA, respectively.

Abbreviation: FMSL, fish meal substitution level.

^a^Feed efficiency (FE) = [total final weight of fish (g) − total initial weight of fish (g) + total weight of dead fish (g)]/total FC (g).

^b^Protein efficiency ratio (PER) = WG of fish (g)/protein consumption of fish (g).

^c^Protein retention (PR, %) = protein gain of fish (g) × 100/protein consumption of fish (g).

^d^Condition factor (CF, g/cm^3^) = body weight of fish (g) × 100/total length of fish (cm)^3^.

^e^Viscerosomatic index (VSI, %) = viscera weight of fish (g) × 100/body weight of fish (g).

^f^Hepatosomatic index (HSI, %) = liver weight of fish (g) × 100/body weight of fish (g).

However, FE, PER, and PR of red sea bream ranged from 1.02–1.04, 1.97–2.02, and 32.17%–33.10%, respectively, but none of these parameters were significantly (*p* > 0.05) influenced by either FMSL or JMM inclusion.

The biological indices (CF, VSI, and HSI) of red sea bream were not significantly (*p* > 0.05) influenced by FMSL or JMM inclusion.

**Figure 1:**
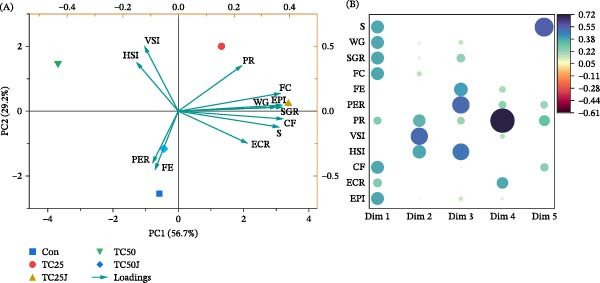
Principal component analysis (PCA) of red sea bream fed the experimental diets (Con, TC25, TC50, TC25J, and TC50J) for 56 days on studied variables including growth, feed utilization, biological indices, and economic analysis; showing PCA biplot of first two principal components (A) and contribution plot of studied parameters on extracted each dimension of principal components (B). Parameters include growth (S: survival, WG: weight gain, and SGR: specific growth rate), feed utilization (FC: feed consumption, FE: feed efficiency, PER: protein efficiency ratio, PR: protein retention), biological indices (VSI: viscerosomatic index, HSI: hepatosomatic index, CF: condition factor), and economic analysis (ECR: economic conversion ratio, and EPI: economic profitability index). Bubble size represents the magnitude of contribution, and color indicates the direction.

The PCA biplot of the growth, feed utilization, biological indices, and economic parameters showed that the Con and TC50J diet group is situated just in the opposite quadrants of the TC25 and TC25J diet groups (Figure 1A). In the biplot, the TC50 diet group is situated far from the other groups in a different quadrant. The Con diet is close to the TC50J diet, while the TC25 is close to the TC25J diet. The PC1 (56.7%) and PC2 (29.2%) collectively explain 85.9% of the total variation. The survival (S), WG, SGR, FC, CF, and EPI have a strong positive influence or equal contribution in PC1 (Figure 1B).

### 3.4. Blood Chemistry of Red Sea Bream

Plasma and serum parameters of fish, except for AST and ALT, were not significantly (*p* > 0.05) affected by FMSL or JMM inclusion level. The low‐FM diets (54.8 ± 0.67 and 8.4 ± 0.23 U/L) with JMM inclusion showed significantly (*p* < 0.028 and *p* < 0.032) lower AST and ALT values than those (57.8 ± 0.70 and 9.2 ± 0.14 U/L, respectively) without JMM inclusion (Table 6). Nevertheless, none of the blood chemistry of fish was significantly (*p* > 0.05) affected by dietary treatments.

**Table 6:** Blood chemistry of red sea bream fed the experimental diets for 56 days.

Experimental diets	Plasma parameters								Serum parameters	
	AST (U/L)	ALT (U/L)	ALP (U/L)	T‐BIL (U/L)	T‐CHO (mg/dL)	TG (mg/dL)	TP (g/dL)	ALB (g/dL)	SOD (ng/mL)	LYZ activity (U/mL)
Con	55.4 ± 2.19	8.6 ± 0.19	200.2 ± 1.42	1.0 ± 0.14	249.1 ± 4.04	213.0 ± 9.43	3.6 ± 0.04	1.0 ± 0.06	1.7 ± 0.07	80.5 ± 12.50
TC25	56.9 ± 1.25	9.0 ± 0.19	210.6 ± 7.33	1.0 ± 0.13	253.3 ± 2.52	224.2 ± 7.18	3.7 ± 0.10	0.9 ± 0.06	2.1 ± 0.38	104.3 ± 9.82
TC50	57.9 ± 0.78	9.3 ± 0.19	208.7 ± 9.67	0.9 ± 0.11	259.1 ± 6.95	221.2 ± 8.84	3.5 ± 0.21	0.8 ± 0.13	2.0 ± 0.29	98.8 ± 5.20
TC25J	54.1 ± 1.25	8.4 ± 0.40	205.8 ± 3.96	0.9 ± 0.11	263.2 ± 6.28	224.8 ± 7.75	3.8 ± 0.16	1.0 ± 0.21	1.9 ± 0.16	109.8 ± 22.13
TC50J	55.4 ± 0.48	8.3 ± 0.33	212.4 ± 5.78	1.1 ± 0.20	250.6 ± 14.58	216.7 ± 4.02	3.8 ± 0.23	1.0 ± 0.13	1.8 ± 0.09	84.7 ± 16.05
*p*‐Value	*p* > 0.356	*p* > 0.155	*p* > 0.687	*p* > 0.887	*p* > 0.703	*p* > 0.782	*p* > 0.687	*p* > 0.749	*p* > 0.884	*p* > 0.566
Major effect: FMSL										
25%	55.5 ± 1.01	8.7 ± 0.23	208.2 ± 3.88	1.0 ± 0.08	258.3 ± 3.75	224.5 ± 4.72	3.8 ± 0.09	0.9 ± 0.10	2.0 ± 0.19	107.1 ± 10.89
50%	56.7 ± 0.68	8.8 ± 0.28	210.6 ± 5.11	1.0 ± 0.11	254.8 ± 7.47	218.9 ± 4.46	3.6 ± 0.15	0.9 ± 0.09	1.9 ± 0.14	91.8 ± 8.18
Major effect: JMM inclusion										
Without	57.4 ± 0.70^A^	9.2 ± 0.14^A^	209.6 ± 5.44	1.0 ± 0.08	256.2 ± 3.55	222.7 ± 5.14	3.6 ± 0.11	0.8 ± 0.07	2.0 ± 0.21	101.6 ± 5.12
With	54.8 ± 0.67^B^	8.4 ± 0.23^B^	209.1 ± 3.47	1.0 ± 0.11	256.9 ± 7.64	220.7 ± 4.30	3.8 ± 0.12	1.0 ± 0.11	1.9 ± 0.09	97.3 ± 13.46
Two‐way ANOVA										
FMSL	*p* > 0.270	*p* > 0.698	*p* > 0.743	*p* > 0.809	*p* > 0.704	*p* > 0.462	*p* > 0.482	*p* > 0.741	*p* > 0.707	*p* > 0.329
JMM inclusion	*p* < 0.028	*p* < 0.032	*p* > 0.943	*p* > 0.809	*p* > 0.943	*p* > 0.786	*p* > 0.384	*p* > 0.335	*p* > 0.617	*p* > 0.776
Interaction	*p* > 0.870	*p* > 0.477	*p* > 0.558	*p* > 0.347	*p* > 0.322	*p* > 0.732	*p* > 0.722	*p* > 0.584	*p* > 0.900	*p* > 0.524

*Note:* Con: the 60% fish meal (FM) basal diet; TC25: dietary substitution of 25%‐FM with TC; TC50: dietary substitution of 50%‐FM with TC; TC25J, dietary substitution of 25%‐FM with TC with JMM inclusion; TC50J, dietary substitution of 50%‐FM with TC with JMM inclusion. FMSL, fish meal substitution level. Values (means of triplicate ± standard error) with different uppercase letters in the column indicate statistical differences (*p* < 0.05) based on two‐way ANOVA followed by Duncan’s multiple range test.

Abbreviations: ALB, albumin; ALP, alkaline phosphatase; ALT, alanine aminotransferase; AST, aspartate aminotransferase; LYZ, lysozyme activity; SOD, superoxide dismutase; T‐BIL, total bilirubin; T‐CHO, total cholesterol; TG, triglyceride; TP, total protein.

### 3.5. Biochemical Composition of the Whole‐Body Red Sea Bream

The moisture content of the whole‐body fish ranged from 69.5% to 71.0%, crude protein ranged from 16.4% to 17.0%, crude lipid ranged from 6.8% to 7.3%, and ash content ranged from 4.4% to 4.5% (Table 7). However, neither FMSL nor JMM inclusion significantly (*p* > 0.05) altered the proximate composition of fish.

**Table 7:** Proximate composition (%, wet weight basis) of the whole‐body red sea bream fed the experimental diets for 56 days.

Experimental diets	Proximate composition (%)			
	Moisture	Crude protein	Crude lipid	Ash
Con	69.8 ± 0.41	16.4 ± 0.10	7.3 ± 0.23	4.4 ± 0.25
TC25	69.9 ± 0.48	17.0 ± 0.19	7.1 ± 0.44	4.4 ± 0.22
TC50	71.0 ± 0.41	16.6 ± 0.27	6.9 ± 0.37	4.4 ± 0.27
TC25J	69.5 ± 0.85	16.8 ± 0.33	6.9 ± 0.23	4.5 ± 0.20
TC50J	70.7 ± 0.44	16.5 ± 0.20	6.8 ± 0.14	4.4 ± 0.23
*p*‐Value	*p* > 0.319	*p* > 0.494	*p* > 0.776	*p* > 0.998
Major effect: FMSL				
25%	69.7 ± 0.44	16.9 ± 0.18	7.0 ± 0.22	4.5 ± 0.13
50%	70.8 ± 0.27	16.6 ± 0.15	6.9 ± 0.18	4.4 ± 0.16
Major effect: JMM inclusion				
Without	70.4 ± 0.37	16.8 ± 0.17	7.0 ± 0.26	4.4 ± 0.16
With	70.1 ± 0.50	16.6 ± 0.18	6.9 ± 0.12	4.5 ± 0.14
Two‐way ANOVA				
FMSL	*p* > 0.082	*p* > 0.287	*p* > 0.662	*p* > 0.830
JMM inclusion	*p* > 0.604	*p* > 0.509	*p* > 0.669	*p* > 0.819
Interaction	*p* > 0.956	*p* > 0.863	*p* > 0.943	*p* > 0.950

*Note:* Con: the 60% fish meal (FM) basal diet; TC25: dietary substitution of 25%‐FM with TC; TC50: dietary substitution of 50%‐FM with TC; TC25J, dietary substitution of 25%‐FM with TC with JMM inclusion; TC50J, dietary substitution of 50%‐FM with TC with JMM inclusion. Values (means of triplicate ± standard error) are presented.

Abbreviation: FMSL, fish meal substitution level.

Neither FMSL nor JMM inclusion had a significant (*p* > 0.05) influence on the amino acid profiles of fish (Table 8).

**Table 8:** Amino acid profiles (% of wet weight) of red sea bream fed the experimental diets for 56 days.

Parameters	Experimental diets					*p*‐Value	Major effect: FMSL		Major effect: JMM inclusion		Two‐way ANOVA		
	Con	TC25	TC50	TC25J	TC50J		25%	50%	Without	With	FMSL	JMM inclusion	Interaction
Essential amino acids (EAA)													
Arginine	0.95 ± 0.020	0.93 ± 0.026	0.90 ± 0.020	0.93 ± 0.032	0.89 ± 0.029	*p* > 0.588	0.93 ± 0.018	0.90 ± 0.016	0.92 ± 0.016	0.91 ± 0.021	*p* > 0.300	*p* > 0.812	*p* > 0.812
Histidine	0.35 ± 0.029	0.35 ± 0.026	0.33 ± 0.026	0.39 ± 0.023	0.39 ± 0.020	*p* > 0.433	0.37 ± 0.017	0.36 ± 0.020	0.34 ± 0.017	0.39 ± 0.014	*p* > 0.789	*p* > 0.088	*p* > 0.594
Isoleucine	0.56 ± 0.029	0.54 ± 0.023	0.53 ± 0.020	0.53 ± 0.026	0.51 ± 0.020	*p* > 0.634	0.53 ± 0.016	0.52 ± 0.014	0.54 ± 0.014	0.52 ± 0.015	*p* > 0.571	*p* > 0.401	*p* > 0.775
Leucine	1.08 ± 0.026	1.08 ± 0.026	1.12 ± 0.026	1.07 ± 0.026	1.10 ± 0.026	*p* > 0.665	1.08 ± 0.017	1.11 ± 0.018	1.10 ± 0.019	1.09 ± 0.017	*p* > 0.259	*p* > 0.501	*p* > 0.757
Lysine	1.24 ± 0.026	1.21 ± 0.020	1.17 ± 0.020	1.19 ± 0.026	1.17 ± 0.020	*p* > 0.242	1.20 ± 0.015	1.17 ± 0.013	1.19 ± 0.015	1.18 ± 0.015	*p* > 0.257	*p* > 0.768	*p* > 0.768
Phenylalanine	0.59 ± 0.023	0.59 ± 0.020	0.62 ± 0.026	0.58 ± 0.026	0.60 ± 0.017	*p* > 0.735	0.59 ± 0.015	0.61 ± 0.015	0.61 ± 0.017	0.59 ± 0.014	*p* > 0.274	*p* > 0.574	*p* > 0.672
Threonine	0.68 ± 0.023	0.67 ± 0.020	0.62 ± 0.032	0.66 ± 0.026	0.63 ± 0.020	*p* > 0.420	0.66 ± 0.015	0.63 ± 0.017	0.64 ± 0.020	0.65 ± 0.016	*p* > 0.182	*p* > 0.897	*p* > 0.609
Tryptophan	0.11 ± 0.012	0.11 ± 0.015	0.10 ± 0.012	0.12 ± 0.015	0.09 ± 0.012	*p* > 0.653	0.11 ± 0.009	0.10 ± 0.008	0.10 ± 0.008	0.10 ± 0.010	*p* > 0.240	*p* > 0.999	*p* > 0.468
Valine	0.67 ± 0.029	0.66 ± 0.017	0.62 ± 0.032	0.66 ± 0.026	0.63 ± 0.017	*p* > 0.637	0.66 ± 0.014	0.63 ± 0.016	0.64 ± 0.018	0.64 ± 0.015	*p* > 0.222	*p* > 0.946	*p* > 0.840
Non‐essential amino acids (NEAA)													
Alanine	1.09 ± 0.020	1.09 ± 0.023	1.12 ± 0.020	1.10 ± 0.017	1.09 ± 0.015	*p* > 0.867	1.10 ± 0.013	1.11 ± 0.012	1.10 ± 0.015	1.10 ± 0.010	*p* > 0.614	*p* > 0.736	*p* > 0.408
Aspartic acid	1.43 ± 0.029	1.42 ± 0.026	1.40 ± 0.032	1.39 ± 0.023	1.38 ± 0.020	*p* > 0.657	1.41 ± 0.017	1.39 ± 0.017	1.41 ± 0.019	1.39 ± 0.014	*p* > 0.534	*p* > 0.390	*p* > 0.707
Glutamic acid	2.10 ± 0.026	2.11 ± 0.032	2.13 ± 0.026	2.10 ± 0.038	2.14 ± 0.032	*p* > 0.905	2.11 ± 0.022	2.13 ± 0.019	2.12 ± 0.019	2.12 ± 0.023	*p* > 0.430	*p* > 0.920	*p* > 0.840
Glycine	1.24 ± 0.032	1.21 ± 0.038	1.20 ± 0.046	1.18 ± 0.026	1.18 ± 0.043	*p* > 0.739	1.20 ± 0.022	1.19 ± 0.029	1.21 ± 0.027	1.18 ± 0.023	*p* > 0.804	*p* > 0.514	*p* > 0.934
Proline	0.74 ± 0.026	0.76 ± 0.020	0.78 ± 0.026	0.75 ± 0.032	0.76 ± 0.020	*p* > 0.907	0.76 ± 0.017	0.77 ± 0.015	0.77 ± 0.015	0.76 ± 0.017	*p* > 0.654	*p* > 0.654	*p* > 0.949
Serine	0.69 ± 0.026	0.68 ± 0.038	0.64 ± 0.020	0.67 ± 0.020	0.64 ± 0.026	*p* > 0.474	0.68 ± 0.019	0.64 ± 0.015	0.66 ± 0.022	0.65 ± 0.016	*p* > 0.193	*p* > 0.765	*p* > 0.765
Tyrosine	0.43 ± 0.020	0.44 ± 0.023	0.42 ± 0.020	0.42 ± 0.032	0.39 ± 0.026	*p* > 0.610	0.43 ± 0.018	0.41 ± 0.017	0.43 ± 0.014	0.41 ± 0.020	*p* > 0.329	*p* > 0.329	*p* > 0.707

*Note:* Con: the 60% fish meal (FM) basal diet; TC25: dietary substitution of 25%‐FM with TC; TC50: dietary substitution of 50%‐FM with TC; TC25J, dietary substitution of 25%‐FM with TC with JMM inclusion; TC50J, dietary substitution of 50%‐FM with TC with JMM inclusion. Values (means of triplicate ± standard error) are presented.

Abbreviation: FMSL, fish meal substitution level.

The 25%‐FM substitution diets led to significantly (*p* < 0.008) higher palmitic acid (C16:0) than the 50%‐FM substitution diets, and the low‐FM diets with JMM inclusion resulted in significantly (*p* < 0.0001) lower palmitic acid than those without JMM inclusion (Table 9). Myristic acid (C14:0) of fish fed the Con diet was significantly (*p* < 0.015) higher than that of fish fed the TC50, TC25J, and TC50J diets. Palmitic acid fed the Con diet was significantly (*p* < 0.0001) higher than that of fish fed all other diets. The low‐FM diets with JMM inclusion led to significantly (*p* < 0.010) lower stearic acid (C18:0) than those without JMM inclusion. Stearic acid of fish fed the TC50J diet was significantly (*p* < 0.003) higher than that of fish fed the Con and TC25 diets.

**Table 9:** Fatty acid profiles (% of total fatty acids) of red sea bream fed the experimental diets for 56 days.

Parameters	Experimental diets					*p*‐Value	Major effect: FMSL		Major effect: JMM inclusion		Two‐way ANOVA		
	Con	TC25	TC50	TC25J	TC50J		25%	50%	Without	With	FMSL	JMM inclusion	Interaction
C14:0	2.12 ± 0.049^a^	1.98 ± 0.061^ab^	1.94 ± 0.032^b^	1.89 ± 0.035^b^	1.86 ± 0.032^b^	*p* < 0.015	1.94 ± 0.038	1.90 ± 0.027	1.96 ± 0.032	1.88 ± 0.022	*p* > 0.445	*p* > 0.070	*p* > 0.876
C16:0	14.24 ± 0.066^a^	14.01 ± 0.040^b^	13.90 ± 0.043^bc^	13.78 ± 0.026^c^	13.63 ± 0.043^d^	*p* < 0.0001	13.90 ± 0.055^A^	13.76 ± 0.066^B^	13.95 ± 0.037^A^	13.71 ± 0.042^B^	*p* < 0.008	*p* < 0.0001	*p* > 0.593
C18:0	5.19 ± 0.069^c^	5.34 ± 0.043^bc^	5.41 ± 0.040^ab^	5.48 ± 0.043^ab^	5.57 ± 0.046^a^	*p* < 0.003	5.41 ± 0.040	5.49 ± 0.045	5.38 ± 0.030^B^	5.52 ± 0.035^A^	*p* > 0.102	*p* < 0.010	*p* > 0.766
∑SFA^a^	21.54 ± 0.052^a^	21.34 ± 0.144^ab^	21.25 ± 0.052^b^	21.15 ± 0.104^b^	21.06 ± 0.029^b^	*p* < 0.024	21.24 ± 0.090	21.16 ± 0.050	21.29 ± 0.071	21.11 ± 0.052	*p* > 0.374	*p* > 0.079	*p* > 0.986
C16:1n‐7	3.38 ± 0.090^a^	3.25 ± 0.032^a^	2.94 ± 0.023^b^	3.30 ± 0.046^a^	3.08 ± 0.029^b^	*p* < 0.001	3.28 ± 0.027^A^	3.01 ± 0.035^B^	3.10 ± 0.072^B^	3.19 ± 0.055^A^	*p* < 0.0001	*p* < 0.024	*p* > 0.202
C17:1n‐7	0.41 ± 0.026	0.38 ± 0.020	0.34 ± 0.026	0.36 ± 0.023	0.33 ± 0.020	*p* > 0.184	0.37 ± 0.015	0.34 ± 0.015	0.36 ± 0.017	0.35 ± 0.015	*p* > 0.178	*p* > 0.481	*p* > 0.775
C18:1n‐9	24.91 ± 0.309	25.42 ± 0.084	25.48 ± 0.061	25.55 ± 0.075	25.63 ± 0.066	*p* > 0.051	25.49 ± 0.058	25.56 ± 0.052	25.45 ± 0.048	25.59 ± 0.049	*p* > 0.349	*p* > 0.091	*p* > 0.875
C20:1n‐9	2.74 ± 0.061^a^	2.55 ± 0.038^b^	2.51 ± 0.043^bc^	2.38 ± 0.040^cd^	2.33 ± 0.064^d^	*p* < 0.002	2.46 ± 0.045	2.42 ± 0.052	2.53 ± 0.027^A^	2.36 ± 0.035^B^	*p* > 0.369	*p* < 0.007	*p* > 0.918
C22:1n‐9	0.36 ± 0.026	0.37 ± 0.017	0.40 ± 0.020	0.38 ± 0.017	0.44 ± 0.026	*p* > 0.139	0.38 ± 0.011^B^	0.42 ± 0.017^A^	0.39 ± 0.014	0.41 ± 0.020	*p* < 0.047	*p* > 0.258	*p* > 0.486
C24:1n‐9	1.69 ± 0.038^a^	1.50 ± 0.032^b^	1.43 ± 0.032^b^	1.52 ± 0.026^b^	1.48 ± 0.035^b^	*p* < 0.003	1.51 ± 0.019	1.46 ± 0.023	1.47 ± 0.025	1.50 ± 0.022	*p* > 0.107	*p* > 0.317	*p* > 0.681
∑MUFA^b^	33.49 ± 0.121	33.48 ± 0.159	33.11 ± 0.006	33.49 ± 0.182	33.30 ± 0.015	*p* > 0.179	33.49 ± 0.108^A^	33.21 ± 0.043^B^	33.30 ± 0.109	33.40 ± 0.092	*p* < 0.048	*p* > 0.425	*p* > 0.486
C18:2n‐6	28.17 ± 0.133^c^	29.98 ± 0.292^a^	30.10 ± 0.188^a^	28.98 ± 0.384^b^	29.54 ± 0.092^ab^	*p* < 0.001	29.48 ± 0.311	29.82 ± 0.156	30.04 ± 0.157^A^	29.26 ± 0.217^B^	*p* > 0.230	*p* < 0.018	*p* > 0.423
C18:3n‐3	2.96 ± 0.043^c^	3.00 ± 0.049^bc^	3.08 ± 0.029^ab^	3.11 ± 0.023^ab^	3.15 ± 0.032^a^	*p* < 0.019	3.05 ± 0.035	3.12 ± 0.025	3.04 ± 0.032^B^	3.13 ± 0.020^A^	*p* > 0.105	*p* < 0.027	*p* > 0.579
C18:3n‐6	0.56 ± 0.038^c^	0.64 ± 0.020^abc^	0.72 ± 0.020^a^	0.60 ± 0.020^bc^	0.68 ± 0.023^ab^	*p* < 0.011	0.62 ± 0.016^B^	0.70 ± 0.016^A^	0.68 ± 0.021	0.64 ± 0.022	*p* < 0.007	*p* > 0.106	*p* > 0.939
C20:4n‐6	1.74 ± 0.090	1.72 ± 0.026	1.65 ± 0.026	1.74 ± 0.026	1.68 ± 0.023	*p* > 0.645	1.73 ± 0.017^A^	1.67 ± 0.017^B^	1.69 ± 0.022	1.71 ± 0.020	*p* < 0.045	*p* > 0.384	*p* > 0.899
C20:5n‐3	4.36 ± 0.029^a^	3.58 ± 0.040^b^	3.50 ± 0.026^bc^	3.40 ± 0.043^cd^	3.34 ± 0.020^d^	*p* < 0.0001	3.49 ± 0.048	3.42 ± 0.039	3.54 ± 0.028^A^	3.37 ± 0.026^B^	*p* > 0.058	*p* < 0.001	*p* > 0.812
C22:2n–6	0.41 ± 0.038	0.38 ± 0.023	0.35 ± 0.020	0.40 ± 0.020	0.37 ± 0.032	*p* > 0.578	0.39 ± 0.014	0.36 ± 0.017	0.36 ± 0.016	0.38 ± 0.018	*p* > 0.229	*p* > 0.473	*p* > 0.947
C22:6n–3	4.03 ± 0.061^c^	4.07 ± 0.035^c^	4.10 ± 0.032^bc^	4.22 ± 0.018^ab^	4.23 ± 0.028^a^	*p* < 0.012	4.14 ± 0.037	4.17 ± 0.034	4.09 ± 0.022^B^	4.22 ± 0.015^A^	*p* > 0.474	*p* < 0.002	*p* > 0.697
∑n‐3 HUFA^c^	8.39 ± 0.032^a^	7.65 ± 0.075^b^	7.60 ± 0.058^b^	7.62 ± 0.057^b^	7.56 ± 0.032^b^	*p* < 0.0001	7.64 ± 0.043	7.58 ± 0.031	7.63 ± 0.044	7.59 ± 0.032	*p* > 0.381	*p* > 0.578	*p* > 0.955
Unknown	2.75 ± 0.188	1.82 ± 0.084	2.15 ± 0.136	2.91 ± 0.681	2.66 ± 0.185	*p* > 0.193	2.37 ± 0.392	2.40 ± 0.153	1.98 ± 0.102	2.79 ± 0.321	*p* > 0.925	*p* > 0.058	*p* > 0.444

*Note:* Con: the 60% fish meal (FM) basal diet; TC25: dietary substitution of 25%‐FM with TC; TC50: dietary substitution of 50%‐FM with TC; TC25J, dietary substitution of 25%‐FM with TC with JMM inclusion; TC50J, dietary substitution of 50%‐FM with TC with JMM inclusion. Values (means of triplicate ± standard error) with different uppercase and lowercase letters in the row indicate statistical differences (*p* < 0.05) based on two‐way ANOVA and Duncan’s multiple range test, respectively.

Abbreviation: FMSL, fish meal substitution level.

^a^∑SFA: total saturated fatty acids.

^b^∑MUFA: total monounsaturated fatty acids.

^c^∑n‐3 HUFA: total n‐3 highly unsaturated fatty acids.

The 25%‐FM substitution diets led to significantly (*p* < 0.048) higher ∑MUFA than the 50%‐FM substitution diets. Nevertheless, there was no significant difference in ∑MUFA among dietary treatments. The 25%‐FM substitution diets led to significantly (*p* < 0.0001) higher palmitoleic acid (C16:1n‐7) than the 50%‐FM substitution diets, while the low‐FM diets with JMM inclusion led to significantly (*p* < 0.024) lower palmitoleic acid than those without JMM inclusion. Palmitoleic acid of fish fed the Con, TC25, and TC25J diets was significantly (*p* < 0.001) higher than that of fish fed the TC50 and TC50J diets. The low‐FM diets with JMM inclusion led to significantly (*p* < 0.007) lower gondoic acid (C20:1n‐9) than those without JMM inclusion. The 25%‐FM substitution diets led to significantly (*p* < 0.047) lower erucic acid (C22:1n‐9) than the 50%‐FM substitution diets.

The low‐FM diets with JMM inclusion led to significantly (*p* < 0.018 and *p* < 0.027, respectively) lower linoleic acid (C18:2n‐6) and higher linolenic (C18:3n‐3) than those without JMM inclusion. Linoleic acid of fish fed the TC25 and TC50 diets was significantly (*p* < 0.001) higher than that of fish fed the Con and TC25J diets. Linolenic acid of fish fed the TC50J diet was significantly (*p* < 0.019) higher than that of fish fed the Con and TC25 diets. The 25%‐FM substitution diets led to significantly (*p* < 0.007) lower gamma‐linolenic acid (C18:3n‐6) than the 50%‐FM substitution diets. The gamma‐linolenic acid of fish fed the TC50 diet was significantly (*p* < 0.011) higher than that of fish fed the Con and TC25J diets. The 25%‐FM substitution diets led to significantly (*p* < 0.045) higher arachidonic acid (C20:4n‐6) than the 50%‐FM substitution diets. The low‐FM diets with JMM inclusion led to significantly (*p* < 0.001 and *p* < 0.002, respectively) higher eicosapentaenoic acid (EPA, C20:5n‐3) and lower docosahexaenoic acid (DHA, C22:6n‐3) than those without JMM inclusion. The EPA of fish fed the Con diet was significantly (*p* < 0.0001) higher than that of fish fed all other diets. DHA of fish fed the TC50J diet was significantly (*p* < 0.012) higher than that of fish fed the Con, TC25, and TC50 diets. In multiple comparisons, ∑n‐3 HUFA of fish fed the Con diet was significantly (*p* < 0.0001) higher than that of fish fed all other diets.

**Figure 2:**
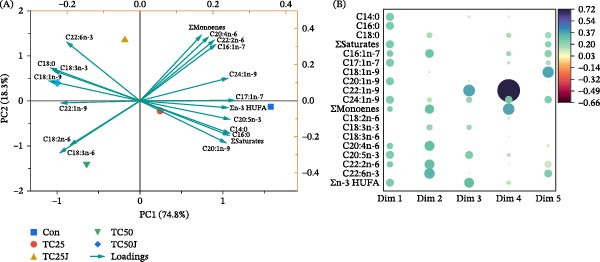
Principal component analysis (PCA) of red sea bream fed the experimental diets (Con, TC25, TC50, TC25J, and TC50J) for 56 days on fatty acid profile; showing PCA biplot of first two principal components (A) and contribution plot of fatty acids on each extracted dimension of principal components (B). Bubble size represents the magnitude of contribution, and color indicates the direction. Here: C14:0, myristic acid; C16:0, palmitic acid; C18:0, stearic acid; C16:1n‐7, palmitoleic acid; C17:1n‐7, heptadecenoic acid; C18:1n‐9, oleic acid; C20:1n‐9, gonodoic acid; C22:1n‐9, erucic acid; C24:1n‐9, nervonic acid; C18:2n‐6, linoleic acid; C18:3n‐3, alpha‐linolenic acid; C18:3n‐6, gamma linolenic acid; C20:4n‐6, arachidonic acid; C20:5n‐3, eicosapentaenoic acid; C22:n‐6, docosadienoic acid; and C22:6n‐3, docosahexaenoic acid.

The PCA biplot of fatty acid profiles showed the Con diet positioned far away from the TC25, TC50, TC25J, and TC50J diet groups (Figure 2A). The PC1 (74.8%) and PC2 (18.3%) comprised 93.1% of total variance. According to the contribution plot (Figure 2B), C22:1n‐9, C18:2n‐6, and C18:3n‐6 contributed neither in PC1 nor PC2, and the rest contributed either individually or both in PC1 and PC2.

### 3.6. Economic Analysis of the Study

The 25%‐FM substitution diets exhibited significantly (*p* < 0.0001) higher ECR than the 50%‐FM substitution diets, while the low‐FM diets with JMM inclusion exhibited significantly (*p* < 0.003) higher ECR than those without JMM (Table 10). ECR of fish fed the Con and TC25J diets was significantly (*p* < 0.0001) higher than that of fish fed all other diets.

**Table 10:** Economic analysis (diet price, economic conversion ratio [ECR] and economic profit index [EPI]) of the feeding trial for 56 days.

Experimental diets	Diet price (USD/kg)	ECR^a^ (USD/kg)	EPI^b^ (USD/fish)
Con	1.85	1.78 ± 0.023^a^	1.03 ± 0.018
TC25	1.67	1.63 ± 0.005^b^	1.05 ± 0.009
TC50	1.49	1.44 ± 0.023^d^	1.01 ± 0.012
TC25J	1.76	1.72 ± 0.014^a^	1.06 ± 0.012
TC50J	1.58	1.51 ± 0.024^c^	1.04 ± 0.009
*p*‐Value	—	*p* < 0.0001	*p* > 0.071
Major effect: FMSL			
25%	—	1.67 ± 0.021^A^	1.06 ± 0.007^A^
50%	—	1.48 ± 0.021^B^	1.02 ± 0.010^B^
Major effect: JMM inclusion			
Without	—	1.54 ± 0.043^B^	1.03 ± 0.012
With	—	1.61 ± 0.049^A^	1.05 ± 0.009
Two‐way ANOVA			
FMSL	—	*p* < 0.0001	*p* < 0.011
JMM inclusion	—	*p* < 0.003	*p* > 0.080
Interaction	—	*p* > 0.540	*p* > 0.371

*Note:* Con: the 60% fish meal (FM) basal diet; TC25: dietary substitution of 25%‐FM with TC; TC50: dietary substitution of 50%‐FM with TC; TC25J, dietary substitution of 25%‐FM with TC with JMM inclusion; TC50J, dietary substitution of 50%‐FM with TC with JMM inclusion. Values (means of triplicate ± standard error) with different lowercase and uppercase letters indicate statistical differences (*p* < 0.05) based on Duncan’s multiple range test and two‐way ANOVA, respectively.

Abbreviation: FMSL, fish meal substitution level.

^a^Economic conversion ratio (ECR, USD/kg) = FC of fish (kg/fish)/WG (kg/fish) × diet price (USD/kg).

^b^Economic profit index (EPI, USD/fish) = [final weight (kg/fish) × fish sale price (USD/kg)] − [ECR (USD/kg) × WG (kg/fish)].

The 25%‐FM substitution diets exhibited significantly (*p* < 0.011) higher EPI than the 50%‐FM substitution diets, but no significant (*p* > 0.05) difference in EPI was found among dietary treatments.

## 4. Discussion

Higher FM substitution with TC in diets led to inferior growth performance (WG and SGR) of red sea bream probably resulted from reduced FC in the present study were coincident with other studies [14, 58, 59], in which the growth of fish tended to decrease with increased dietary FM substitution with the alternative sources. In general, increased FM replacement with an alternative source commonly deteriorated the palatability of low‐FM diets, reduced FC by fish, and eventually brought about decreased WG [22, 60]. Incorporating a feed enhancer or feed stimulant in low‐FM diets could resolve these issues. Therefore, JMM, exhibiting the strongest feeding behavior response to red sea bream, was also applied as a feed enhancer in low‐FM diets, substituting 25% and 50% FM with TC for red sea bream in this study. Improvement in WG and SGR of red sea bream receiving low‐FM diets with JMM inclusion compared to those without JMM inclusion agreed with other studies [61, 62] showing that incorporating a feed enhancer or feed stimulant in low‐FM diets effectively improved the growth of fish.

TBM has the potential to replace FM up to 75% in the diets for rockfish [32] and 50% for olive flounder [28], and 25%–30% for spotted rose snapper [31] without negatively impacting growth and FC. In particular, 40% of FM could be replaced with TBM in a 55% FM‐based diet for red sea bream without significantly impairing growth performance and FC [34]. The substitutability of FM with CGM in diets has been reported to be 10%, 30%, and 40% in Asian seabass [36], two‐banded sea bream (*Diplodus vulgaris*) [63], and olive flounder [40], respectively, without adversely affecting growth performance and feed conversion. Our earlier study [44] proved that 20% of FM could be substituted with CGM in a 55% FM‐based diet of red sea bream without significantly lowering growth performance, FC, and feed utilization in a 56‐day feeding trial.

No significant differences in WG, SGR, FC, and feed utilization were observed between red sea bream fed the Con and TC50 diets in the present experiment. These results suggest that up to 50% of FM can be substituted with TC in a 55% FM‐based diet without compromising growth performance, FC, and feed utilization. This finding may also imply potential synergistic effects of FM substitution with TC, considering that TBM and CGM can individually replace up to 40% [34] and 20% of FM [44], respectively, without significantly impairing growth and feed utilization. Therefore, FM replacement with TC provided greater potential for synergistic effects than a single source, in terms of nutritional balance, the possibility of incorporating higher levels in diets, as well as cost‐effectiveness and availability.

Furthermore, the inclusion of JMM in low‐FM diets (TC25J and TC50J diets) improved growth performance and FC of red sea bream compared to those fed the Con diet in this experiment. In addition, superior WG and SGR of red sea bream fed the TC25J and TC50J diets compared to those fed the TC25 and TC50 diets, respectively, suggested that JMM inclusion in the low‐FM diets could enhance diet palatability and FC, thereby contributing to improved growth. This improvement could be attributed to the stronger preference of red sea bream for JMM over other protein sources [49].

A single source of either animal or plant protein often requires supplementation with EAA in the diets of red sea bream [18, 64]. In contrast, combining animal and plant protein sources can exert synergistic effects that mitigate the nutritional deficiencies or imbalances inherent in individual ingredients [23, 24]. This synergy enhances the efficacy of the TC in replacing FM, as opposed to using TBM or CGM alone. For instance, TBM contains relatively low levels of most EAAs, except for histidine and tryptophan, when compared to FM, whereas CGM is rich in leucine and phenylalanine. The combination of these proteins (TC) yields higher leucine and phenylalanine levels than FM, a pattern clearly observed in the TC‐substituted diets (TC25 and TC50 diets) without JMM inclusion in the present study. These findings demonstrate the high potential of TC as an FM replacer in red sea bream diets, primarily due to its synergistic nutritional composition.

Specifically, a 5% FM‐based diet supplemented with 0.54% leucine led to enhanced muscle fiber growth and development in blunt snout bream (*Megalograma amblycephala*) compared to that without leucine supplementation [65]. Normally, phenylalanine serves as a precursor for tyrosine [66]. Supplementation of phenylalanine in diets improved growth performance and FE in silver perch (*Bidyanus bidyanus*) [67] and Indian major carp (*Cirrhinus mrigala*) [68]. Furthermore, a diet containing a low (3.4 g/kg) phenylalanine level resulted in inferior growth performance and feed intake in grass carp (*Ctenopharyngodon idella*) compared to diets containing higher phenylalanine levels (6.1, 9.1, 11.5, 14.0, and 16.8 g/kg) [69]. Although the requirements of these indispensable amino acids (leucine and phenylalanine) remain unknown for red sea bream, the low‐FM diets substituting 25% and 50%‐FM with TC may have influenced the levels of EAAs, particularly leucine and phenylalanine. This potential alteration was reflected in the comparable FC and may have contributed slightly, though not significantly, to lower or higher WG and SGR in fish fed the TC25 and TC50 diets compared to those fed the Con diet. Furthermore, the inclusion of JMM in the low‐FM diets increased the histidine content, which may have contributed to improving FC and consequently resulted in a significant improvement in SGR in this study [70].

Increased FM substitution with TC in this study also resulted in elevated levels of NEAAs, particularly alanine, glutamic acid, and proline in diets. These changes may have contributed to improving FC in fish fed the TC25, TC25J, and TC50J diets. L‐alanine has been identified as a potent feeding stimulant in channel catfish (*Ictalurus punctatus*), eliciting consummatory behaviors such as turning, increased gill pumping, and biting or snapping even at concentrations as low as 10^−6^ M [71]. Similarly, glutamic acid is also recognized as a potential feeding stimulant in fish, particularly carnivorous species, by enhancing feed palatability and promoting voluntary feed intake [72].

Normally, both EAA and NEAA are crucial for protein synthesis and the physiological function of fish, and EAA must be supplied through diets, while NEAA can be synthesized by fish [73]. An alternative source with or without supplementation of EAA for FM in fish diets commonly limits their substitutability [74, 75]. Although dietary requirements for most of the EAAs in red sea bream remain unknown, except for arginine [55], lysine [56], and valine [57], requirements for these known EAAs were fulfilled in all experimental diets in the current study. Minor variations in EAA content among the low‐FM diets are unlikely to have adversely affected the growth performance of red sea bream.

Carnivorous fish require n‐3 HUFA [76], including EPA and DHA in their diets due to their limited biosynthetic capacity and these fatty acids are essential for membrane integrity, immune regulation, and neural function, thereby ensuring optimal growth and survival [77, 78]. Furthermore, SGR of golden gray mullet (*Liza aurata*) was significantly lower when fed a diet containing a low (0.2%) level of n‐3 HUFA compared to those fed a diet containing a standard level (1.2%) of n‐3 HUFA [79]. The optimal DHA:EPA ratio in fish diets varied among fish species [80]; however, this ratio remained undetermined for red sea bream. In the present study, the ∑n‐3 HUFA tended to decrease with increased FM substitution in the low‐FM diets, regardless of 24% JMM inclusion, but the DHA‐to‐EPA ratio tended to increase compared to the Con diet. Although the reduced ∑n‐3 HUFA did not appear to negatively impact the growth performance of fish, a relatively higher DHA‐to‐EPA ratio (0.96 and 1.14) in the TC25J and TC50J diets may have contributed to improved growth [81]. Although the National Research Council [82] reported that the optimal DHA:EPA ratio for marine fish ranges between 0.5 and 2.0, further research is recommended to determine the precise dietary requirement for red sea bream.

The 25%‐FM‐substitution diets led to higher FC than the 50%‐FM substitution diets in this experiment, agreeing with other studies [14, 22, 58], in which dietary increased FM substitution with an alternative protein source led to decreased FC by fish, ascribed to reduced palatability and acceptability of low‐FM diets [14, 83]. Likewise, FC of red sea bream fed the 25%‐FM‐replacement diets was higher than that of fish fed the 50%‐FM‐replacement diets [84].

Slightly, but not significantly, improved FC in the TC25 diet or decreased FC in the TC50 diet compared to the Con diet led to slightly, but not significantly, improved or decreased growth performance in red sea bream, respectively. Furthermore, significantly improved FC in the TC25J diet or slightly, but not significantly, improved FC in the TC50J diet compared to the Con diet led to significantly improved or slightly improved WG and SGR in red sea bream, respectively. This might imply that the growth performance of red sea bream was directly reflected by FC. No significant differences in FE, PER, and PR of fish in this experiment might demonstrate the proportional growth performance to FC. The current study also demonstrates that JMM inclusion in the low‐FM diets could improve FC of red sea bream and eventually enhance WG and SGR. Baek and Cho [49] reported that JMM was identified as the most appealing ingredient among 18 protein feed ingredients for red sea bream, and its inclusion in the diet effectively improved FC and growth performance.

According to the PCA biplot, the Con, TC50, and TC50J diets align closely, the same as for TC25 and TC25J diets, indicating similar biological responses. This suggests that 50% of FM substitution with TC, regardless of JMM inclusion in the low‐FM diet, appears to yield similar results; thereby, 50% of FM may be substituted with TC without deteriorating the evaluated biological parameters. Moreover, the TC25J diet appeared to be the best‐performing diet based on the growth, feed utilization, biological indices, and economic analysis, as it mostly influenced the pure growth‐related parameters.

Substitutability of various animal and plant protein sources and their combination have been reported in red sea bream diets [14, 18, 33, 34, 44, 64, 84–88] (Table 11). Substitutability of animal protein sources for FM in red sea bream diets has been reported to be 20%–50% and 20%–75% for plant protein sources. In particular, up to 20% and 40% of FM could be replaced with CGM [44] and TBM [34] in diets of red sea bream, respectively. Furthermore, the dietary FM replacement level with TBM could be increased up to 50% with 24% JMM inclusion in the red sea bream diet [84]. FM substitution levels with CGM in red sea bream diets increased with fish size [44, 87]. The combination of animal and plant protein sources (fermented soybean meal and squid by‐product, fermented soybean meal and scallop by‐product, fish soluble, krill meal, squid meal, and dehulled soybean meal) have enabled to improve the FM substitution level up to 80% without any adverse effects on growth and feed utilization [88]. The results observed from these studies highlight the importance of combined protein sources for higher FM substitution in diets of red sea bream, thereby reducing dependences on the expensive and unsustainable FM.

**Table 11:** Overview of FM substitution levels with animal, plant, and their combinations in diets of juvenile red sea bream.

Substitutes	FM (%) levels in the basal diet	Substitution level (%)	Supplemented EAA	Initial weight of fish (g)	Feeding days (frequency/ day)	Recommended levels based on the observed parameters	References
Animal protein sources							
Black soldier fly meal	64	20, 40, 60, 80, and 100	DL‐methionine, L‐lysine HCI	17.9	56 (2)	Up to 40% without compromising growth and feed efficiency.	[18]
Blood meal	60	10, 20, and 30	DL‐methionine	17.5	56 (2)	Up to 20% without adverse growth and feed efficiency	[85]
Meat meal (MM)	55	20, 40, 60, 80, and 100	—	7.8	56 (2)	Up to 40% without compromising growth and FC.	[86]
Tuna by‐product meal (TBM)	55	20, 40, 60, 80, and 100	—	8.6	56 (2)	Up to 40% without reducing growth and feed availability.	[34]
TBM	60	25 and 50	—	11.8	56 (2)	Up to 50% with 24% JMM inclusion without affecting growth, feed utilization, chemical composition, and AA profiles	[84]
Tuna muscle powder	58.5	25, 50, 75, and 100	—	6.6	50 (2)	Up to 50% without affecting growth.	[33]
Plant protein sources							
Corn gluten meal (CGM)	55	20, 40, and 60	—	8.6	56 (2)	Up to 20% without affecting growth, feed availability, blood chemistry, proximate composition, and fatty acid profiles.	[44]
CGM	50	30, 50, 70, 90, and 100	—	53.0	40 (3)	Up to 30% without affecting growth and feed utilization.	[87]
Fermented rapeseed meal	47	18.75, 37.5, 56.25, and 75	Methionine, Lysine	3.5	65 (2)	Up to 56.25% without affecting growth, feed utilization, and immune defense system.	[14]
Soy protein isolate	61	25, 50, and 75	DL‐methionine, L‐lysine	4.1	56 (2)	Up to 75% without compromising growth and feed utilization.	[64]
Combined							
Fermented soybean meal and squid by‐product, fermented soybean meal and scallop by‐product, fish soluble, krill meal, squid meal, and dehulled soybean meal	60	60, 80, and 100	—	1.3	56 (2)	Up to 80% without negative effects on growth, feed utilization, body composition, physiological state, digestibility, and quality of fish.	[88]
TBM, CGM	60	25 and 50	—	2.0	56 (2)	Up to 50% without affecting growth performance, feed utilization, biological indices, blood chemistry, amino acid profiles, and profitability regardless of JMM inclusion.	The present study

*Note:* Fish were fed to apparent satiation in all studies, except for [33] (5% body weight).

Abbreviations: EAA, essential amino acids; FC, feed consumption; FM, Fish meal.

CF is a widely used morphometric indicator that assesses the health status and well‐being of fish [89]. No significant differences in the CF of fish were found in this experiment. In addition, VSI of fish serves as a lipid utilization biomarker, demonstrating a positive correlation with dietary lipid levels [90]. The high‐lipid diets have been shown to increase VSI in gilthead sea bream, indicating greater lipid accumulation [91]. In this study, however, no significant differences in VSI were observed among dietary treatments, likely due to the isolipidic (15.2%) content across all experimental diets. HSI is a widely used indicator of fish nutritional status as it indirectly reflects hepatic glycogen and glucose levels [92]. Diets with elevated carbohydrate levels have frequently resulted in increased HSI, reflecting enhanced hepatic glycogen and carbohydrate deposition in silver sea bream (*Sparus sarba*) [93] and mandarin fish (*Siniperca chuatsi*) [94]. In the present study, however, neither FMSL nor inclusion of JMM significantly influenced the biological indices of fish. Similarly, replacing FM with a combination of seafood by‐products and soybean proteins had no effect on CF or HSI in red sea bream [88].

Fish blood parameters can be used to diagnose disorders and analyze blood damage, offering a reliable sign of the health status of fish [95, 96]. Plasma parameters serve as indicators for evaluating the physiological response of fish to dietary intake, environmental conditions, and overall well‐being [15, 97]. Plasma parameters of fish were not significantly altered by either FMSL or JMM inclusion, except for a main effect of JMM inclusion on AST and ALT levels. The significantly lower plasma AST and ALT levels observed in fish fed low‐FM diets with JMM inclusion compared to those without JMM inclusion might be attributed to the high digestibility and balanced amino acid profiles in JMM. This might reduce hepatic metabolic load along with its n‐3 HUFA level, which enhances membrane stability and reduces inflammatory stress [98], thereby reducing hepatic enzyme leakage. This indicates improved liver function in red sea bream. In a previous study, plasma parameters of red sea bream fed diets in which FM was replaced with a combination of soybean meal, CGM, and meat meal remained within normal physiological range; however, fish fed an FM‐based diet exhibited a more stable physiological state than those fed FM‐replaced diets [6].

In fish, innate immunity is the first line of defense system that can be used to analyze the effects of nutrition on immune function and health [99, 100]. Lysozyme, a bacteria‐lysing enzyme, is found throughout the body and is essential to most animals’ inherent defensive mechanisms [99, 100]. SOD is an antioxidant enzyme that protects cells from oxidative damage by catalyzing the dismutation of superoxide radicals [101, 102]. In the current study, serum SOD and LYZ activity were not significantly affected by either FMSL or JMM inclusion in the low‐FM diets [51].

Neither FMSL [103] nor JMM inclusion in the low‐FM diets affected the whole‐body proximate composition and amino acid profiles of red sea bream in this study. Similarly, supplementation of 20% feed attractant (10% fish soluble, 5% krill meal, and 5% squid meal) in diets replacing 70%, 80%, 90%, and 100% of FM with dehulled soybean meal did not affect the proximate composition of red sea bream [48]. In general, fish can maintain a relatively stable whole‐body amino acid profile despite moderate variation in dietary composition within EAA requirements, primarily by regulating internal amino acid levels through metabolic pathways [103, 104]. Notably, muscle protein synthesis rate accounts for only about 20% of the whole‐body protein synthesis in fish [105], which may help to explain why the whole‐body amino acid profiles remained largely unaffected by dietary modification in this study, indicating that overall dietary protein and lipid content seemed to be appropriate.

Typically, the fatty acid profiles in fish muscle were largely reflected in the fatty acid profiles of diets [106, 107]. Likewise, the whole‐body fatty acid profiles of fish were affected by the dietary fatty acid profiles in this study. Similarly, dietary fatty acid profiles influenced the fatty acid profiles in the muscle or fillet in Atlantic salmon [106], carp (*Cyprinus carpio*) [107], and Jian carp (*Cyprinus carpio var*. Jian) [108], Nile tilapia (*Oreochromis niloticus*) [109], rainbow trout [110, 111], and red sea bream [112].

According to the PCA biplot, fish fed the Con diet comprise a distinct cluster compared to the low‐FM diets with and without JMM inclusion, suggesting substantial differences in fatty acid profiles of red sea bream fed the experimental diets. Fish fed the TC50 and TC50J diets are moderately associated with elevated levels of linoleic acid and gamma‐linolenic acid, whereas those fed the TC25 and TC25J diets, particularly the TC25J diet, were more closely aligned with increased levels of n‐3 fatty acid, including DHA and alpha‐linolenic acid, as well as ∑MUFA. These findings suggest that the TC25J diet may promote n‐3 fatty acid profiles in red sea bream, while the TC50 and TC50J diets may promote the accumulation of n‐6 fatty acids.

In aquaculture, feed expenses can account for up to 70% of the total costs, with high levels of FM inclusion being a major contribution to these elevated costs [113]. Consequently, reducing FM inclusion through optimized feed formulations represents a strategic approach to lowering feed costs and improving the economic sustainability of aquaculture operations [114, 115]. TBM is recognized as a cost‐effective alternative to FM and a sustainable protein source in aquaculture feed formulation [32, 114]. Although TBM is less expensive than FM, its combination with CGM helps balance not only the nutritional profile but also the overall diet cost. In this experiment, diet prices tended to decrease with increasing FM replacement using TC. However, the inclusion of JMM in low‐FM diets resulted in a slight increase in feed cost. Given the added cost of JMM, a more comprehensive economic assessment is warranted. Nevertheless, moderate inclusion of JMM in low‐FM diets for red sea bream appeared to be economically justifiable [84]. Similarly, ECR decreased with increasing dietary FMSL, indicating improved cost efficiency of the diets. From an economic perspective, however, EPI provides a more comprehensive measure of profitability as it incorporates FC, WG, feed cost, and the market price of fish [83, 116]. In the present study, the 25%‐FM substitution diets exhibited significantly higher EPI values compared to the 50% FM substitution diets. No significant differences in WG, SGR, FC, and EPI between the Con and TC50 diets may indicate that 50% of FM replacement with TC could be commercially feasible in this study. In addition, the inclusion of JMM in the low‐FM diets did not lead to any significant changes in the EPI. Although no statistically significant differences in EPI were observed among all dietary treatments, the TC25J diet yielded the highest numerical EPI value, suggesting the greatest potential profitability for farmers.

## 5. Conclusion

The 25%‐FM substitution diets exhibited higher WG, SGR, FC, and EPI than the 50%‐FM substitution diets. Inclusion of JMM in the low‐FM diet further enhanced WG and SGR. However, feed utilization, biological indices, proximate composition, and amino acid profiles of fish were not significantly affected by either FMSL or JMM inclusion in the low‐FM diets. Overall, TC can effectively replace up to 50% of FM in a 60% FM‐based diet, regardless of JMM inclusion, without negatively impacting growth performance, FC, feed utilization, proximate composition, amino acid profiles, or economic profitability in red sea bream. Among all treatments, the TC25J diet was the most favorable, showing improved growth performance and FC, and the highest EPI, thereby offering the greatest economic benefit to farmers.

## Author Contributions

**Jabed Hasan:** Writing – original draft, visualization, formal analysis, data curation, investigation, methodology, conceptualization. **Sung Hwoan Cho:** Writing – review & editing, visualization, project administration, funding acquisition, supervision, resources, validation, methodology, conceptualization. **Hee Sung Kim:** Data curation, funding acquisition.

## Funding

This work was supported by the National Research Foundation of Korea grant funded by the Korean Government (No. 2020R1A2C1009903). This research was supported by the Korea Institute of Marine Science & Technology Promotion (KIMST) funded by the Ministry of Oceans and Fisheries (02263222).

## Ethics Statement

All experimental protocols followed Ethical Principles and Guidelines for Scientific Animal Use and received approval from the Institutional Animal Care and Use Committee (IACUC) of Korea Maritime and Ocean University (Busan, Korea) (KMOU IACUC 2021‐04).

## Conflicts of Interest

The authors declare no conflicts of interest.

## Data Availability

Data are available upon request from the authors.
